# mTORC1 Activation in Osteoclasts Prevents Bone Loss in a Mouse Model of Osteoporosis

**DOI:** 10.3389/fphar.2019.00684

**Published:** 2019-06-13

**Authors:** Manami Hiraiwa, Kakeru Ozaki, Takanori Yamada, Takashi Iezaki, Gyujin Park, Kazuya Fukasawa, Tetsuhiro Horie, Hikari Kamada, Kazuya Tokumura, Mei Motono, Katsuyuki Kaneda, Eiichi Hinoi

**Affiliations:** ^1^Laboratory of Molecular Pharmacology, Division of Pharmaceutical Sciences, Kanazawa University Graduate School, Kanazawa, Japan; ^2^Venture Business Laboratory, Organization of Frontier Science and Innovation, Kanazawa University, Kanazawa, Japan

**Keywords:** metabolic bone diseases, mTORC1, osteoclastogenesis, Raptor, tuberous sclerosis complex 1

## Abstract

The mechanistic/mammalian target of rapamycin (mTOR) is widely implicated in the pathogenesis of various diseases, including cancer, obesity, and cardiovascular disease. Bone homeostasis is maintained by the actions of bone-resorbing osteoclasts and bone-forming osteoblasts. An imbalance in the sophisticated regulation of osteoclasts and osteoblasts leads to the pathogenesis as well as etiology of certain metabolic bone diseases, including osteoporosis and osteopetrosis. Here, we identified mTOR complex 1 (mTORC1) as a pivotal mediator in the regulation of bone resorption and bone homeostasis under pathological conditions through its expression in osteoclasts. The activity of mTORC1, which was indicated by the phosphorylation level of its downstream target p70S6 kinase, was reduced during osteoclast differentiation, in accordance with the upregulation of Hamartin (encoded by tuberous sclerosis complex 1 [*Tsc1*]), a negative regulator of mTORC1. Receptor activator of nuclear factor-κB ligand (RANKL)-dependent osteoclastogenesis was impaired in *Tsc1*-deficient bone marrow macrophages. By contrast, osteoclastogenesis was markedly enhanced by *Raptor* deficiency but was unaffected by *Rictor* deficiency. The deletion of *Tsc1* in osteoclast lineage cells in mice prevented bone resorption and bone loss in a RANKL-induced mouse model of osteoporosis, although neither bone volume nor osteoclastic parameter was markedly altered in these knockout mice under physiological conditions. Therefore, these findings suggest that mTORC1 is a key potential target for the treatment of bone diseases.

## Introduction

The mechanistic/mammalian target of rapamycin (mTOR) is a kinase belonging to the phosphoinositide 3-kinase-related kinase (PIKK) family of protein kinase. mTOR is composed of two forms of distinct complexes designated as mTOR complex 1 (mTORC1) and mTORC2 (Brown et al., 1994; Sabatini et al., 1994). It is widely implicated in various cellular functions such as growth, proliferation, survival, autophagy, differentiation, and cytoskeletal organization (Sabatini, 2017). The Raptor subunit belongs to mTORC1. Tuberous sclerosis complex 1 (*Tsc1*) and complex 2 (*Tsc2*), which encode Hamartin and Tuberin, respectively, are critical negative regulators of mTORC1 through their GTPase-activating protein activity toward the small G-protein Ras homolog enriched in the brain (Nojima et al., 2003; Long et al., 2005). On the contrary, the Rictor subunit belongs to mTORC2 (Jacinto et al., 2004). Although global deletion of *mTOR*, *Raptor*, and *Rictor* results in embryonic lethality, cell-specific deletion strategies show that mTOR is implicated in the pathogenesis of various diseases, including cancer, obesity, and cardiovascular disease (Murakami et al., 2004; Guertin et al., 2006; Shiota et al., 2006).

Mesenchymal cells differentiate into skeletal elements by forming a cartilaginous model, which induces bone formation through endochondral ossification in the vertebral column and long bones (Karsenty et al., 2009). Endochondral ossification is required for proper skeletal development and bone modeling, while skeleton integrity, as well as bone remodeling, is believed to be coordinately regulated by two different types of cells, bone-forming osteoblasts and bone-resorbing osteoclasts (Harada and Rodan, 2003; Teitelbaum and Ross, 2003). An imbalance in the sophisticated regulation of osteoclasts and osteoblasts leads to pathogenesis as well as the etiology of certain metabolic bone diseases such as osteoporosis, osteopetrosis, and rheumatoid arthritis (Feng and McDonald, 2011).

Studies on genetic mouse have revealed a critical role of mTORC1 in skeletal development through its expression in mesenchymal stem cells or chondrocytes (Chen and Long, 2014; Yan et al., 2016). Additionally, our recent study showed the critical role of mTORC1 in skeletogenesis through the translational control of *Sox9* RNA in mesenchymal stem cells (Iezaki et al., 2018). In addition to its role in skeletal development, mTORC1 is essential for the maintaining bone homeostasis through its expression in bone-forming osteoblasts and bone-resorbing osteoclasts (Chen and Long, 2018). Although several independent lines of evidence based on pharmacological and genetic strategies show that mTOR signal is important for osteoclast differentiation and function in vitro and in vivo, the precise role of mTOR in osteoclastogenesis is controversial and unknown (Chen et al., 2015; Dai et al., 2017; Zhang et al., 2017; Chen and Long, 2018; Huynh and Wan, 2018).

## Materials and Methods

### Materials

Glutathione S-transferase (GST)-receptor activator of nuclear factor-κB ligand (RANKL) vector and PLAT-E cells were obtained from Dr. S.L. Teitelbaum (Washington University, St. Louis, MO, USA) and T. Kitamura (Tokyo University, Tokyo, Japan), respectively. pMSCVpuro-Cre (#34564) was obtained from Addgene (Watertown, MA, USA). Recombinant mouse RANKL and macrophage colony-stimulating factor (M-CSF) were purchased from R&D Systems (Minneapolis, MN, USA). C-terminal Peptide of Type I Collagen (CTx) Enzyme-linked immunosorbent assay (ELISA) kit was obtained from Immunodiagnostic Systems (Boldon, UK). Antibodies were from the following companies: anti-β-actin was from Santa Cruz Biotechnology (Santa Cruz, CA, USA); anti-mTOR, anti-Raptor, anti-Rictor, anti-Hamartin, anti-p-p70S6K1, and anti-p70S6K1 were from Cell Signaling Technology (Danvers, MA, USA). THUNDERBIRD SYBR quantitative polymerase chain reaction (qPCR) Mix was supplied by TOYOBO (Osaka, Japan). Other chemicals used were all of the highest purity commercially available.

### Mice

The protocol used here meets the guideline of the Japanese Society for Pharmacology and was approved by the Committee for Ethical Use of Experimental Animals at Kanazawa University. *Raptor^fl/fl^*, *Rictor^fl/fl^*, and *Tsc1^fl/fl^* mice were obtained from Jackson laboratory. *Tsc1^fl/fl^* mice were crossed with *Lyz2-Cre* mice to generate *Lyz2-Cre;Tsc1^fl/+^* mice, and the resulting progenies were intercrossed to obtain *Lyz2-Cre;Tsc1^fl/fl^* mice. These mutant mice were backcrossed more than five generations with C57BL/6J. Mice were bred under standard animal housing conditions at 23 ± 1°C with humidity of 55% and a light/dark cycle of 12 h, with free access to food and water. Genotyping was performed by PCR using tail genomic DNA. The numbers of animals used per experiment are stated in the figure legends.

For generation of an osteoclast-activated osteoporosis model mouse, 8-week-old mice were intraperitoneally administrated GST–RANKL fusion protein daily for 2 days. Mice were killed by decapitation under deep anesthesia with chloral hydrate (400 mg/kg, intraperitoneal injection) 12 h after final injection (Tomimori et al., 2009; Iezaki et al., 2016).

### Bone Histomorphometric Analyses

Bone histomorphometric analyses were performed on vertebrae not decalcified as previously described (Yamamoto et al., 2012). Briefly, vertebrae were fixed with 10% formalin, followed by dehydration in different concentrations of ethanol and subsequent embedding in methyl methacrylate resin according to standard protocols. The bone volume to tissue volume ratio (BV/TV) ratio was measured by von Kossa staining. The bone formation rate (BFR) was analyzed by the calcein double-labeling method. Calcein was injected to mice twice with an interval of 3 days, and then mice were killed 2 days after the last injection. Osteoblast and osteoclast parameters were analyzed by staining with toluidine blue and with tartrate-resistant acid phosphatase (TRAP), respectively. Analyses were performed using the OsteoMeasure Analysis System (OsteoMetrics) according to standard protocols (Hinoi et al., 2007).

### Retroviral Transfection

Retroviral vectors were transfected into PLAT-E cells using the calcium carbonate method. Virus supernatants were collected 48 h after transfection, and then cells were infected with virus supernatants for 72 h in the presence of 4 μg/mL of polybrene. Cells were then subjected to selection by culture with 1 μg/mL of puromycin for 3 days before usage for experiments (Fukasawa et al., 2016).

### Culture of Osteoclasts and TRAP Staining, the Pit Formation Assay, and the Actin Ring Formation Assay

Bone marrow macrophages (BMMs) were seeded on 48-well plates (Nunc) at a density of 2.5 × 10^4^ cells/well and were cultured in the presence of 20 ng/mL of M-CSF (100 ng/mL of M-CSF for BMMs infected with retrovirus) and 20 ng/mL of RANKL for 4 days consecutively (Wang et al., 2006). TRAP staining, the pit formation assay, and the actin ring formation assay were performed as previously described (Iezaki et al., 2016). Briefly, cells were cultured on bone slices and subsequent fixation with 4% paraformaldehyde, followed by permeabilization with 0.5% Triton X-100. For the pit assay, the bone slices were scrubbed with a brush to remove attached cells. The bone slices were then treated with 20 μg/mL of lectin solution and subsequent incubation with 0.5 mg/mL of 3,3′-diaminobenzidine (DAB), including 0.01% H_2_O_2_.

### Isolation of Osteoclasts

Isolation of osteoclasts was performed from long bones as previously described (Fukasawa et al., 2016). In brief, the long bones were minced into small pieces, and cells were dissociated from bone fragments by vigorous vortex in Alpha modification of Eagle’s MEM (αMEM). After removal of bone fragments by sedimentation under normal gravity, the unfractionated bone cells were seeded on collagen gel and were incubated in αMEM with 5% Fetal bovine serum (FBS). The cells were sequentially treated with 0.001% Pronase E and 0.02% EDTA, 0.01% collagenase, and 0.1% collagenase. Cells released by final treatment were collected and cultured as osteoclasts.

### Real-Time-Based Quantitative PCR

Total RNA was extracted from cells, followed by synthesis of cDNA with reverse transcriptase and oligo-dT primer. The cDNA samples were then used as templates for real-time PCR analysis, which was performed on an Mx3005P instrument (Agilent Technologies), by using specific primers for each gene (**Table 1**). Expression levels of the genes examined were normalized by using the 36b4 expression levels as an internal control for each sample (Nakamura et al., 2013).

**Table 1:** List of primers used for real-time PCR.

Genes	Upstream (5′–3′)	Downstream (5′–3′)
Acp5	CACTCCCACCCTGAGATTTGT	CATCGTCTGCACGGTTCTG
Calcr	GTCACTCCTTGTCGATTGCTG	GTTCCCACTGCATTGTCCACA
Ctsk	GAAGAAGACTCACCAGAAGCAG	TCCAGGTTATGGGCAGAGATT
36b4	GAGGAATCAGATGAGGATATGGGA	AAGCAGGCTGACTTGGTTGC

### Immunoblotting Analysis

Cultured cells were solubilized in lysis buffer containing 1% Nonidet P-40. Samples were then subjected to sodium dodecyl sulfate-polyacrylamide gel electrophoresis (SDS-PAGE), followed by transfer to polyvinylidene difluoride (PVDF) membranes and subsequent immunoblotting assay. Quantification was performed by densitometry using ImageJ.

### Data Analysis

All results are expressed as the mean ± standard error of the mean, and statistical significance was determined using the two-tailed Student *t*-test, and the one-way or two-way ANOVA with Bonferroni/Dunnett *post hoc* test.

## Results

### Expression Level of mTORC1-Related Factors in Primary Cultured Osteoclasts

**Figure 1:**
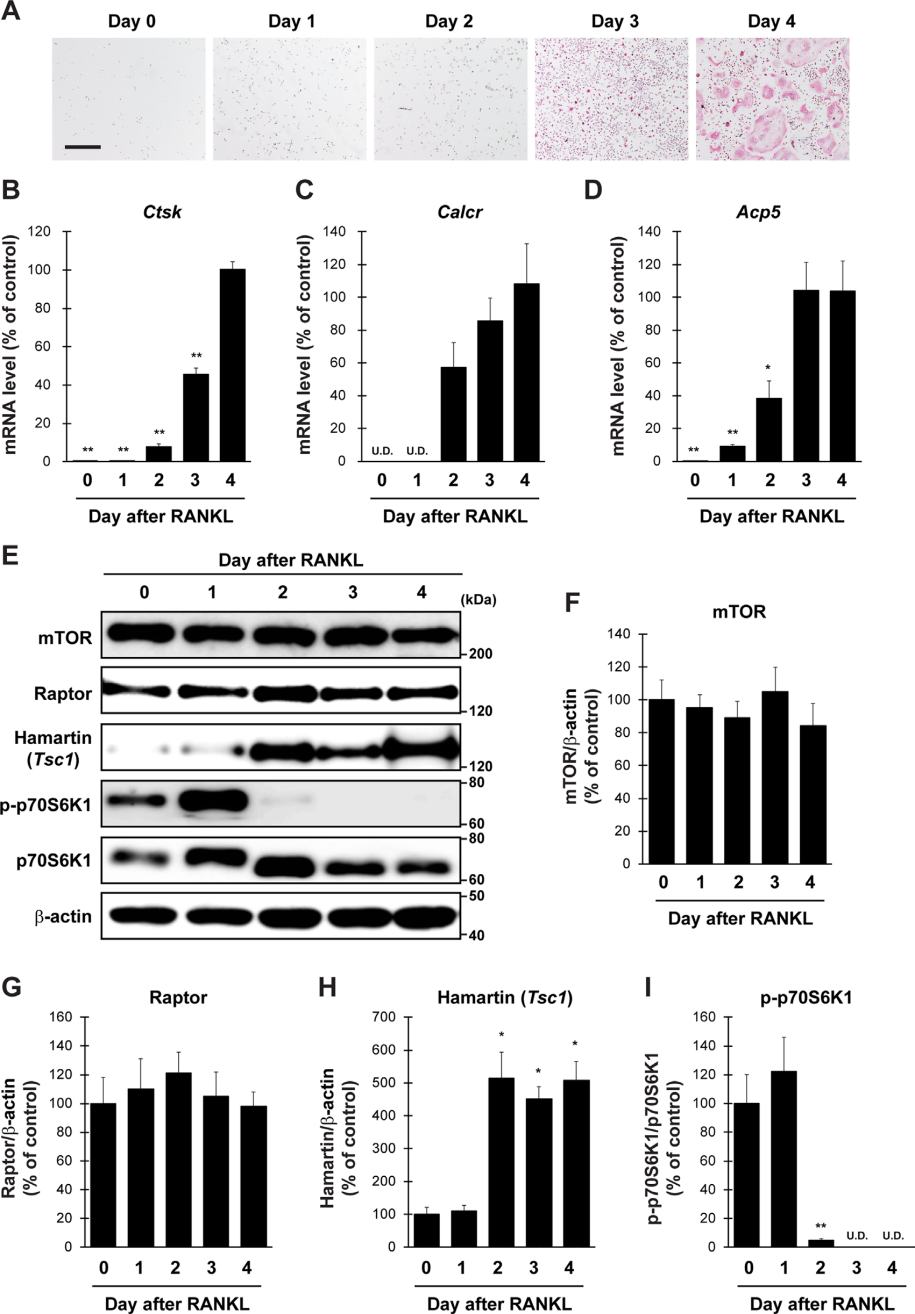
Expression profile of mTORC1-related factors in primary cultured osteoclasts. BMMs prepared from naive mice were stimulated with RANKL and M-CSF, followed by **(A)** TRAP staining and **(B–D)** determination of mRNA expression of osteoclastic marker genes by qPCR at day 0 to day 4 (*n* = 4). **(E–I)** BMMs from naive mice were stimulated with RANKL, followed by determination of protein expression of mTORC1-related factors by immunoblotting at day 0 to day 4 (*n* = 3). All data were analyzed by the one-way ANOVA with Bonferroni/Dunnett *post hoc* test. *P < 0.05, **P < 0.01, significantly different from the value obtained in cells at **(B–D)** day 4 or **(F–I)** day 0. U.D., under detection. Bar = 100 μm.

To evaluate the validity of primary cultured osteoclasts, TRAP staining and qPCR analysis were performed. TRAP-positive cells were increased in proportion to the culture period up to 4 days (**Figure 1A**). qPCR analysis revealed that the expression of all osteoclastic marker genes, including cathepsin K (*Ctsk*), calcitonin receptor (*Calcr*), and acid phosphatase 5 (*Acp5*), was drastically increased during culture from 0 to 4 days (**Figure 1B–D**). We next examined protein level of mTORC1-related factors in cultured osteoclasts (**Figure 1E**). The expression of mTOR and Raptor in osteoclasts was stable throughout the differentiation period (**Figure 1F and G**), whereas the expression of Hamartin (*Tsc1*) was markedly increased in osteoclasts 2 days after RANKL stimulation, and the increase was sustained thereafter (**Figure 1H**). In accordance with the upregulation of Hamartin (*Tsc1*) expression, the phosphorylation level of p70S6 kinase (p70S6K), a downstream effector of mTORC1 signaling, was markedly abolished in osteoclasts 2 days after RANKL stimulation (**Figure 1I**), indicating a possible inverse correlation between Hamartin (*Tsc1*) expression and p70S6K phosphorylation during osteoclastogenesis. These results suggest that mTORC1 activity in vitro is reduced during osteoclast differentiation in a Hamartin (*Tsc1*)-dependent manner.

### Osteoclast Differentiation is Regulated by mTORC1 but not by mTORC2 *In Vitro*

**Figure 2:**
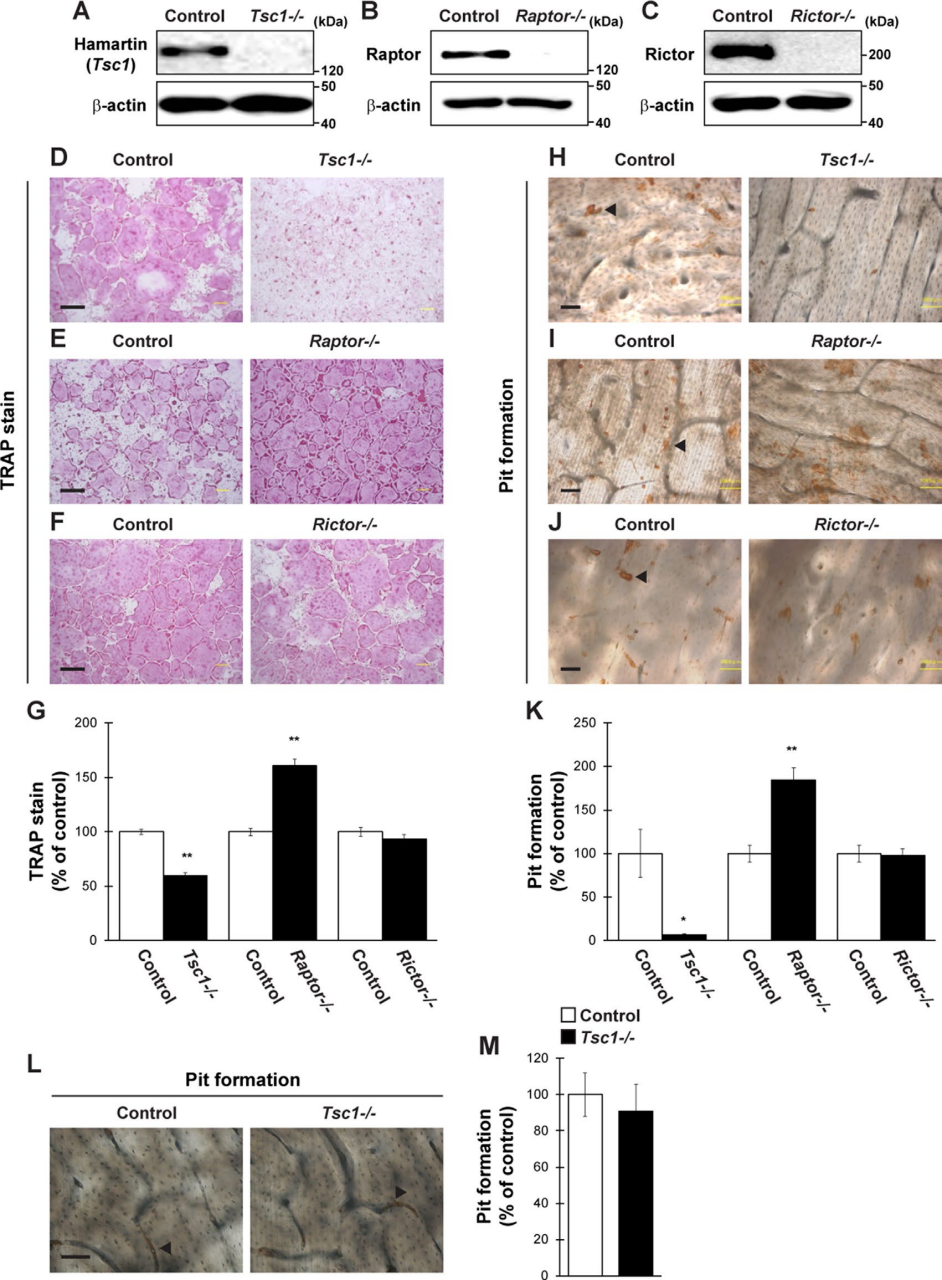
Osteoclastogenesis is regulated by mTORC1 activity. **(A–C)** BMMs from *Tsc1fl/fl* mice, *Raptorfl/fl* mice, or *Rictorfl/fl* mice were retrovirally infected with Cre recombinase, followed by determination of Hamartin (*Tsc1*), Raptor, or Rictor expression (*n* = 3). **(D–K)** BMMs from *Tsc1fl/fl* mice, *Raptorfl/fl* mice, or *Rictorfl/fl* mice were retrovirally infected with Cre recombinase and subsequent stimulation with RANKL, followed by performing **(D–G)** TRAP staining and **(H–K)** pit formation assay (*n* = 3). (**L** and **M**) Osteoclasts prepared from *Tsc1fl/fl* mice were retrovirally infected with Cre recombinase and subsequent pit formation assay (*n* = 3). (**H–J** and **L**) Black arrowheads indicate the representative resorption pit, and the brown staining is due to the DAB staining used in the procedure. All data were analyzed by the two-tailed Student *t*-test. *P < 0.05, **P < 0.01, significantly different from the value obtained in cells infected with empty vector. Bar = 100 μm.

We then evaluated whether the regulators of mTOR pathway control osteoclastogenesis through expression in osteoclasts in vitro. *Tsc1^fl/fl^*, *Raptor^fl/fl^*, and *Rictor^fl/fl^* mice-derived BMMs were infected with a retrovirus expressing Cre recombinase and subjected to differentiation by RANKL, followed by TRAP staining and pit formation assay. The expression of Hamartin (*Tsc1*), Raptor, and Rictor was markedly repressed in retrovirus-infected BMMs (**Figure 2A–C**). The number of TRAP-positive multinucleated cells and area of pit formation were significantly decreased in *Tsc1*-deleted BMMs but significantly increased in *Raptor*-deleted BMMs compared with those in control cells (**Figure 2D, E, G, H, I, K**). By contrast, the number of TRAP-positive multinucleated cells and area of pit formation were comparable between control cells and *Rictor*-deleted BMMs (**Figure 2F, G, J, K**). Moreover, the number of actin rings formed was significantly decreased in *Tsc1*-deleted BMMs (**Supplemental Figure 1A** and **1B**), indicating that mTORC1, rather than mTORC2, controls osteoclast differentiation in a cell-autonomous manner. Conversely, *Tsc1* deficiency did not alter the area of pit formation when the same number of mature osteoclasts was used for pit formation assay (**Figure 2L and M**), suggesting that *Tsc1* regulates osteoclast differentiation rather than maturation *in vitro*.

### Conditional Deletion of *Tsc1* in Osteoclasts Does Not Affect Bone Phenotype Under Normal Physiological Conditions

**Figure 3:**
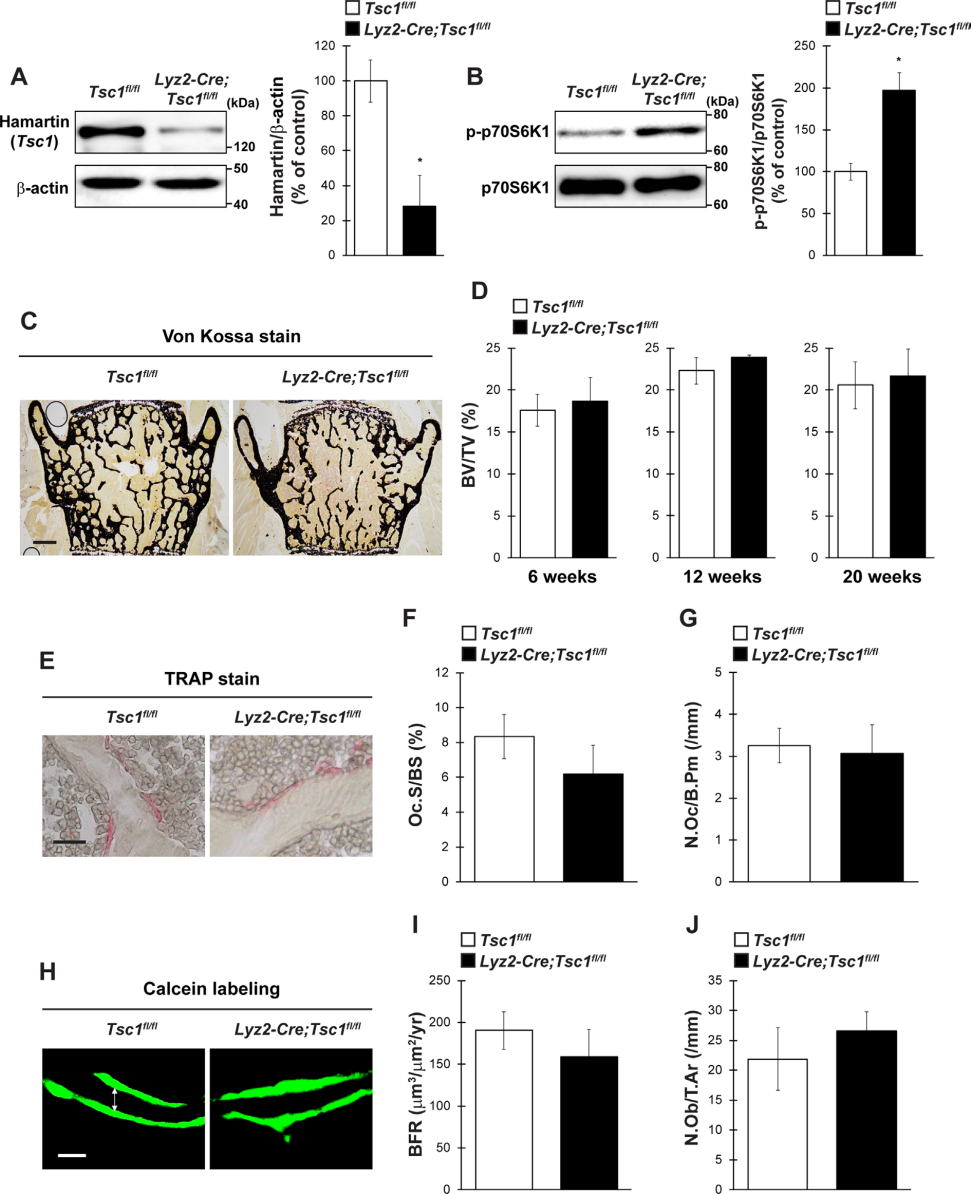
*Tsc1* expressed by osteoclast lineage cells is dispensable for bone resorption and bone mass under physiological condition. **(A)** Hamartin (*Tsc1*) expression in BMMs of control and *Lyz2-Cre;Tsc1fl/fl* mice (*n* = 3). **(B)** Phosphorylation level of p70S6K1 in BMMs of control and *Lyz2-Cre;Tsc1fl/fl* mice (*n* = 3). **(C)** von Kossa staining, **(D)** BV/TV (6-, 12-, and 20-week-old), **(E)** TRAP staining, **(F)** Oc.S/BS, **(G)** N.Oc/B.Pm, **(H)** calcein double labeling, **(I)** BFR, and **(J)** N.Ob/T.Ar of vertebrae of control and *Lyz2-Cre;Tsc1fl/fl* mice at 12-week-old (control, *n* = 5; *Lyz2-Cre;Tsc1fl/fl*, *n* = 6). **(C)** Bar = 200 μm, **(E)** bar = 50 μm, and **(H)** bar = 10 μm. **(H)** Double-headed white arrow indicates the distance between calcein double labeling. All data were analyzed by the two-tailed Student *t*-test.

To evaluate the physiological importance of *Tsc1* in bone homeostasis in vivo, we generated osteoclast lineage cell-specific *Tsc1* knockout mice by crossing *Tsc1^fl/fl^* mice with *Lyz2-Cre* mice. The physical appearance, body weight, and naso-anal length of mice lacking *Tsc1* in Lyz2-expressing cells (hereafter referred to as *Lyz2-Cre;Tsc1^fl/fl^* mice) were normal than in control mice (data not shown). In the BMMs of *Lyz2-Cre;Tsc1^fl/fl^* mice, Hamartin (*Tsc1*) expression was significantly abolished, and the level of phosphorylated p70S6K was significantly increased, indicating an increase in mTORC1 activity in osteoclast lineage cells (**Figure 3A and B**). The BV/TV of *Lyz2-Cre;Tsc1^fl/fl^* mice was indistinguishable from that of control mice in vertebrae at any age examined (**Figure 3C and D**). Bone histomorphometric analyses revealed that indices of osteoclastic function including osteoclast surface (Oc.S/BS) and osteoclast number (N.Oc/B.Pm) (**Figure 3E–G**), in addition to bone formation indices such as BFR and osteoblast number (N.Ob/T.Ar), were comparable between *Lyz2-Cre;Tsc1^fl/fl^* mice and control mice (**Figure 3H–J**). These results show that *Tsc1* deletion in osteoclast lineage cells does not affect bone volume or indices of osteoblastic and osteoclastic function under physiological conditions.

### Conditional Deletion of *Tsc1* in Osteoclasts Prevents RANKL-Induced Osteoclast Activation and Bone Loss

**Figure 4:**
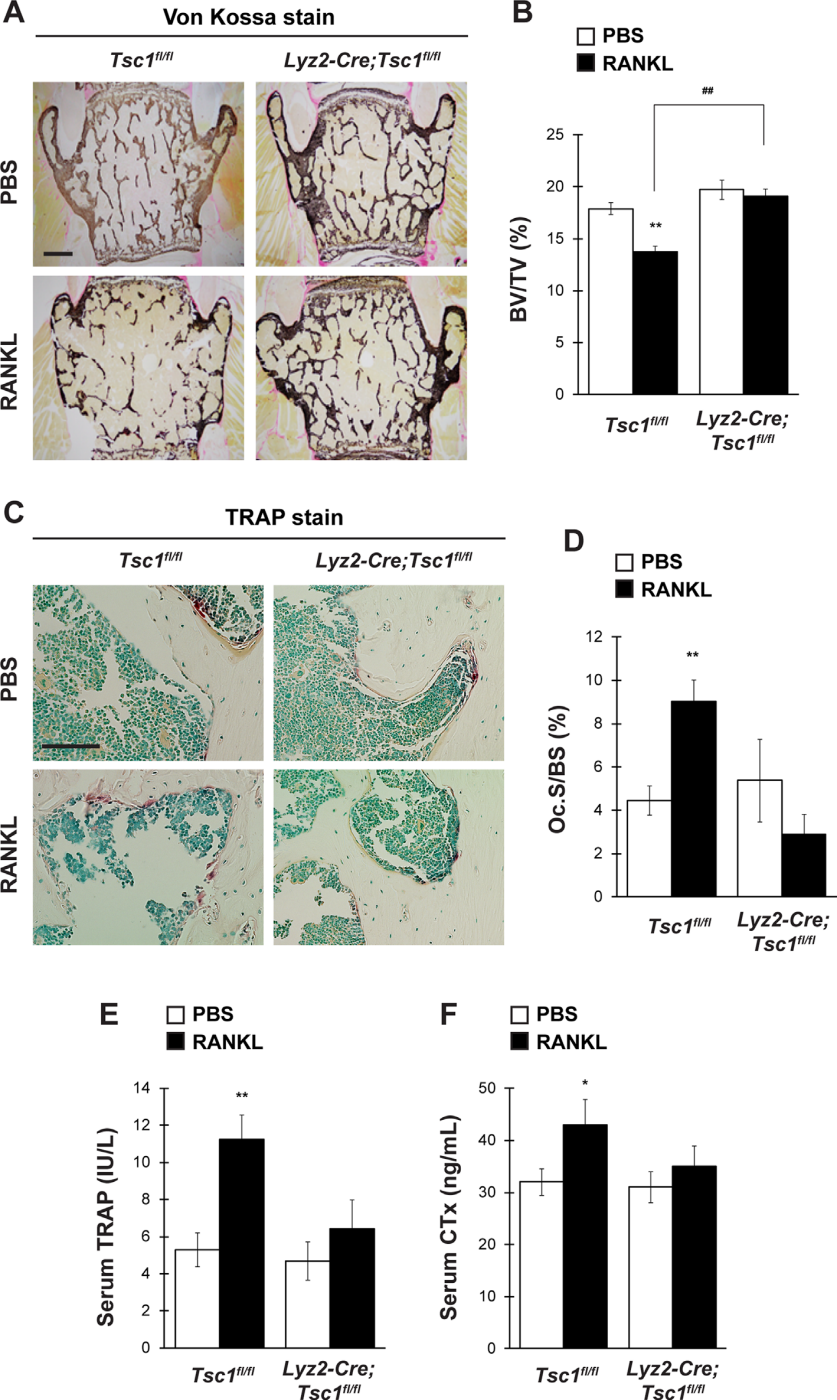
mTORC1 in osteoclast lineage cells is implicated in RANKL-induced bone loss. Control mice and *Lyz2-Cre;Tsc1fl/fl* mice were intraperitoneally administrated with GST–RANKL at 2 mg/kg daily for 2 days, and subsequently, mice were sacrificed 12 h after the final injection, followed by determination of bone phenotypes (control-PBS, *n* = 6; control-RANKL, *n* = 6; *Lyz2-Cre;Tsc1fl/fl*-PBS, *n* = 7; *Lyz2-Cre;Tsc1fl/fl*-RANKL, *n* = 8). **(A)** von Kossa staining, **(B)** BV/TV, **(C)** TRAP staining, and **(D)** Oc.S/BS of the vertebrae of PBS- or RANKL-injected mice. Serum **(E)** TRAP and **(F)** CTx levels of PBS- or RANKL-injected mice. All data were analyzed by the two-way ANOVA with Bonferroni/Dunnett post hoc test. *P < 0.05, **P < 0.01, significantly different from the value obtained for PBS-injected mice.^ ##^ *P* < 0.01 significantly different from the value obtained for RANKL-injected control mice. **(A)** Bar = 200 μm and **(C)** bar = 50 μm.

Osteoclast lineage-specific knockout of *Tsc1* did not cause any abnormalities in bone homeostasis under physiological conditions (**Figure 3**). However, osteoclast differentiation was markedly impaired in *Tsc1*-deficient BMMs (**Figure 2**). This suggests that mTORC1 activation in osteoclasts affects osteoclastogenesis and bone homeostasis under pathological conditions. To test this hypothesis, we investigated the possibility of the involvement of Hamartin (*Tsc1*) expressed by osteoclasts in the pathogenesis of osteoporosis mouse model using GST–RANKL fusion protein injections. RANKL was injected in control and *Lyz2-Cre;Tsc1^fl/fl^* mice daily rate over 2 days, followed by bone phenotype analysis. RANKL injection induced marked bone loss in the vertebrae of control mice but not in those of *Lyz2-Cre;Tsc1^fl/fl^* mice, and RANKL-induced bone loss was markedly impaired in *Lyz2-Cre;Tsc1^fl/fl^* mice (**Figure 4A and B**). RANKL-induced increase in osteoclastic parameter (Oc.S/BS) was observed in control mice but not in *Lyz2-Cre;Tsc1^fl/fl^* mice (**Figure 4C and D**). In addition, RANKL treatment increased the serum TRAP and CTx levels in control mice but not in *Lyz2-Cre;Tsc1^fl/fl^* mice (**Figure 4E and F**), suggesting that Hamartin (*Tsc1*) could be implicated in the osteoclastogenesis and bone resorption in vivo under pathological conditions.

## Discussion

Genetic studies have recently revealed the role of mTORC1 in the cellular function of osteoclasts *in vitro* and bone homeostasis *in vivo*; however, the results of these studies have been inconsistent. Previously, inactivation of mTORC1 signaling in osteoclasts has been shown to increase bone mass in mice by inhibiting osteoclast differentiation due to the deletion of *Raptor* by *Ctsk-Cre*, whereas a decrease in bone mass has been observed by augmenting osteoclast differentiation in mice by deleting *Raptor* with *Lyz2-Cre* (Dai et al., 2017; Zhang et al., 2017). Conversely, *Tsc1* deletion by *Lyz2-Cre* increases bone mass and reduces bone resorption (Zhang et al., 2017; Huynh and Wan, 2018). In addition, conditional deletion of *Tsc1* in hematopoietic stem cells using *Vav1-Cre* results in high bone mass as a consequence of the impairment of osteoclastogenesis (Huynh and Wan, 2018). Contrary to previous findings, we did not observe any bone phenotypes in *Lyz2-Cre;Tsc1^fl/fl^* mice under physiological conditions at any age examined, irrespective of the deletion efficiency (**Figure 3**). The use of different genetic background and housing condition may also contribute to discrepancies between our results and those of previous studies. Nonetheless, our study shows that the serine/threonine kinase mTORC1 regulates bone resorption and bone remodeling under pathological conditions with activated osteoclasts, through its expression in osteoclast lineage cells.

In this study, we showed that osteoclast differentiation and its function in *Rictor*-deficient osteoclasts were indistinguishable from those in control cells, suggesting that mTORC2 does not play a critical role in RANKL-dependent osteoclastogenesis in vitro. Although mTORC2 indirectly promotes osteoclastogenesis by modulating RANKL expression in osteoblasts, there is no evidence supporting the role of mTORC2 in bone resorption and bone homeostasis, through its expression in osteoclast lineage cells in vivo (Chen et al., 2015). An in vitro pharmacological study shows the possibility that mTORC2 regulates osteoclast fusion through Akt signaling (Tiedemann et al., 2017). However, due to the lack of an mTORC2-specific inhibitor and its widespread expression, it is noteworthy to demonstrate the functional relevance of mTORC2 on osteoclastogenesis, bone resorption, and bone remodeling in a cell-specific manner, by generating osteoclast lineage-specific *Rictor*-deficient mice.

It should be also noted that mTORC1 activity was markedly impaired during the course of osteoclast differentiation, in accordance with the marked upregulation of Hamartin (*Tsc1*) detected in this study. It has been shown that RANKL induces direct dephosphorylation of mTOR by calcineurin, leading to the downregulation of mTORC1 activity during osteoclastogenesis (Huynh and Wan, 2018). Our study demonstrated that augmentation of the protein level of a potent mTORC1 inhibitor, Hamartin (*Tsc1*), contributes to the downregulation of mTORC1 activity through RANKL stimulation during the course of osteoclast differentiation. However, further studies are needed to elucidate the exact mechanism(s) underlying RANKL-mediated upregulation of Hamartin (*Tsc1*) in osteoclasts.

In conclusion, the results of this study support the assertion that mTORC1, rather than mTORC2, is functionally expressed in osteoclast lineage cells to repress osteoclast differentiation during bone resorption and remodeling under pathological conditions. These data suggest that mTORC1 signal could be a future target for drug discovery and development for the treatment and therapy of a variety of metabolic bone diseases caused by abnormal activation of osteoclasts, such as osteoporosis, lytic bone metastasis, and rheumatoid arthritis. Moreover, considering that the mTORC1 pathway could be a key modulator of inflammatory conditions such as aging and rheumatic disease (Tomimori et al., 2009; Johnson et al., 2013), our findings may contribute to opportunities for the development of drugs targeting age-related pathologies.

## Ethics Statement

The protocol used here meets the guideline of the Japanese Society for Pharmacology and was approved by the Committee for Ethical Use of Experimental Animals at Kanazawa University.

## Author Contributions

Study design: EH. Study conduct: MH, KO, TY, TI, GP, KF, TH, HK, KT, and MM. Data collection: MH, KO, TY, TI, GP, KF, TH, and HK. Data analysis: MH, TY, TI, KO, GP, KF, TH, and HK. Data interpretation: MH, KO, TY, TI, GP, KF, TH, HK, KK and EH. Drafting manuscript: EH. Approving the final version of the manuscript: All authors.

## Funding

This work was supported in part by the Japan Society for the Promotion of Science (16H05131, 17KT0051, and 18H04971 to EH) and the Japan Agency for Medical Research and Development (17824969 to EH).

## Conflict of Interest Statement

The authors declare that the research was conducted in the absence of any commercial or financial relationships that could be construed as a potential conflict of interest.

## Supplementary Material

Supplemental Figure 1 Actin ring formation assay. BMMs from *Tsc1fl/fl* mice were retrovirally infected with Cre recombinase, and subsequent stimulation with RANKL, followed by actin ring formation assay (*n* = 3). **P*< 0.05, significantly different from the value obtained in control cells.
