# Supplementary figures and images for: mTORC1 Activation in Osteoclasts Prevents Bone Loss in a Mouse Model of Osteoporosis
Source: Front Pharmacol. 2019 Jun 13;10:684. doi: 10.3389/fphar.2019.00684 (PMC6585391; doi:10.3389/fphar.2019.00684)

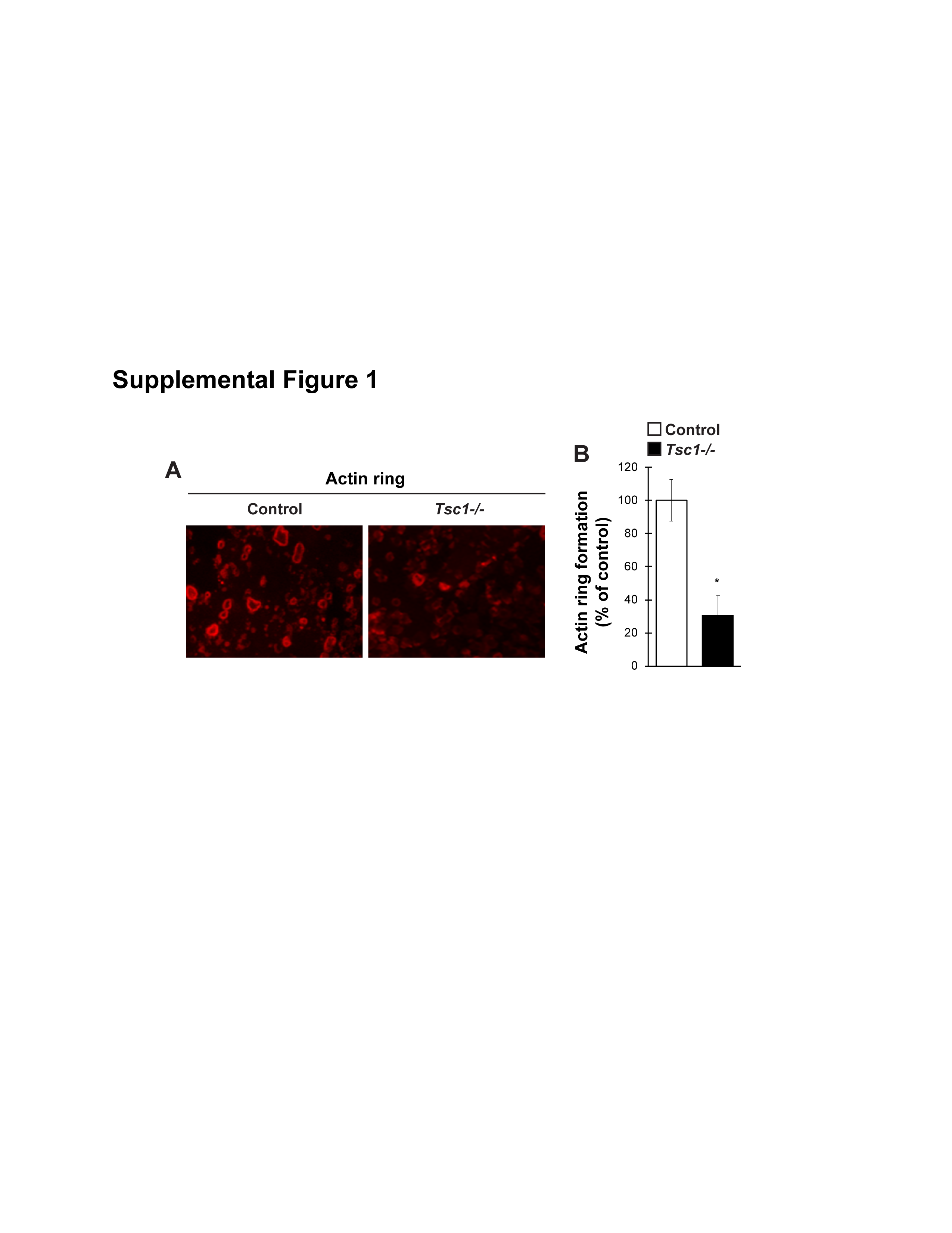

Supplement: Supplemental Figure 1 — Actin ring formation assay. BMMs from Tsc1fl/fl mice were retrovirally infected with Cre recombinase, and subsequent stimulation with RANKL, followed by actin ring formation assay (n = 3). *P< 0.05, significantly different from the value obtained in control cells. [file Image_1.tif]
